# Recent progresses and challenges in aqueous lithium–air batteries relating to the solid electrolyte separator: A mini-review

**DOI:** 10.3389/fchem.2022.1035691

**Published:** 2022-10-10

**Authors:** Peng Chen, Fan Bai, Jun wen Deng, Bin Liu, Tao Zhang

**Affiliations:** ^1^ School of Network and Communication Engineering, Jinling Institute of Technology, Nanjing, China; ^2^ State Key Laboratory of High Performance Ceramics and Superfine Microstructure, Shanghai Institute of Ceramics, Chinese Academy of Sciences, Shanghai, China; ^3^ Department of Applied Chemistry, Kyushu University, Fukuoka, Japan

**Keywords:** Lithium air, Li–O_2_, solid electrolyte, aqueous Li–air, hybrid Li–air

## Abstract

The lithium–air (Li–air) battery utilizes infinite oxygen in the air to store or release energy through a semi-open cathode structure and bears an ultra-high theoretical energy density of more than 1,000 Wh/kg. Therefore, it has been denoted as the candidate for next-generation energy storage in versatile fields such as electric vehicles, telecommunications, and special power supply. Among all types of Li–air batteries, an aqueous Li–air battery bears the advantages of a high theoretical energy density of more than 1,700 Wh/kg and does not have the critical pure oxygen atmosphere issues in a non-aqueous lithium–air battery system, which is more promising for the actual application. To date, great achievements have been made in materials’ design and cell configurations, but critical challenges still remain in the field of the solid electrolyte separator, its related lithium stripping/plating at the lithium anode, and catholyte design. In this mini-review, we summarized recent progress related to the solid electrolyte in aqueous Li–air batteries focusing on both material and battery device development. Moreover, we proposed a discussion and unique outlook on improving solid electrolyte compatibility and battery performance, thus designing an aqueous Li–air battery with higher energy density and better cycle performance in the future.

## Introduction

Modern smart societies depend on the grid of smart devices to facilitate our lives. When almost all smart devices in the modern society need energy to drive and perform, the demand for high energy density energy storage devices has extensively soared in the fields of electric vehicles, consumer electronics, and household appliances (Braun et al., 2012; Shen et al., 2018; Wang et al., 2020a). In contrast, the iteration of energy storage devices gradually fails to keep up with the industry evolution pace as it has been more than 30 years since the commercial announcement of the lithium-ion battery by Sony Corporation (Blomgren, 2016). Under these circumstances, new prototype energy storage systems have been proposed lately hoping to raise the energy storage efficiency and minimize battery weight in the device. Among them, the Li–air battery has been denoted as the holy grail in batteries because of its ultimate theoretical energy density surpassing 3,500 Wh/kg (Abraham and Jiang, 1996). This value is more than 10 times higher than the commercial lithium–ion battery and even comparable to gasoline if the tank-to-wheel energy efficiency of around 13% is considered (Girishkumar et al., 2010).

**FIGURE 1:**
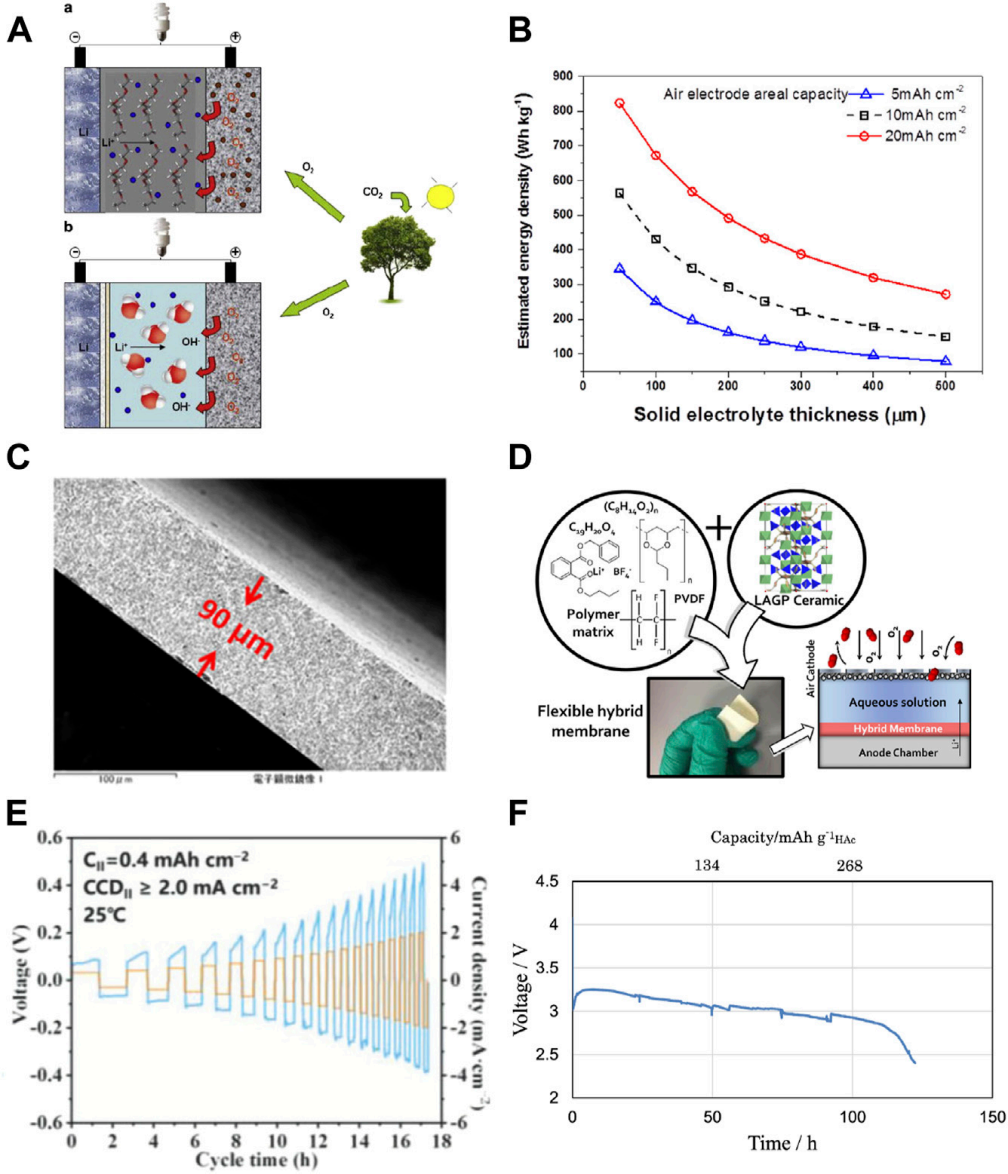
**(A)** Schematic illustration of non-aqueous (upper) and aqueous Li-air batteries (down) [Taken from Capsoni et al. (2012) with permission from Elsevier]; **(B)** the lithium-air battery energy density in variation of LAGTP solid electrolyte thickness. The areal capacity of the electrode is assumed to be 5, 10, and 20 mAh cm^−2^ [Taken from Bai et al. (2019) with permission from Elsevier]; **(C)** cross-sectional image of tape-casted LAGTP pellet [Taken from Bai et al. (2019) with permission from Elsevier]; **(D)** flexible hybrid separator for aqueous lithium air battery [Taken from Safanama and Adams (2017) with permission from ACS]; **(E)** critical current densities of Li/LiF-LiCl-LLZO /Li symmetric cells [Taken from Ruan et al. (2021) with permission from Wiley]; **(F)** discharge curves of Li/KW/Celgard/(LiFSI-2G4)-50 vol% DOL/LAGTP-E/HAc-H2O (1:1 v/v) cell with 0.21 g of HAc at 0.5 mA cm^−2^ and 25°C under an air atmosphere [Taken from Soga et al. (2020) under the terms of the Creative Commons Attribution 4.0 License].

Upon two decades of development, Li–air batteries could mainly be divided into two categories, and they are non-aqueous (Feng et al., 2016; Shu et al., 2019) and aqueous (Zhang et al., 2011; Imanishi et al., 2012; Lu et al., 2014) based on the electrolyte type and oxygen reaction (Capsoni et al., 2012). For the non-aqueous Li–air battery, it possesses an ultra-high theoretical mass energy density of 3,505 Wh/kg and a volume energy density of 3,436 Wh/L (Bruce et al., 2012), derived from a two-electron lithium–oxygen reaction at the cathode side (Eq. 1) when utilizing the organic electrolyte and pure oxygen (Figure 1A). However, until now, no non-aqueous Li–air battery with either high power density or high specific areal capacity has been proposed. Critical challenges still remain in the large decomposition overpotential of lithium peroxide during charge and its clogging at cathode porous channels during discharge (Feng et al., 2016; Imanishi and Yamamoto, 2019). More importantly, the current non-aqueous Li–air battery requires a pure oxygen operation atmosphere, and therefore, eliminating H_2_O and CO_2_ side reactions at both the cathode and anode sides is also crucial (Qiao et al., 2019

$${2Li}^{+}+O_{2}+2e\to {Li}_{2}O_{2}\;E^{0}=2.96\;V,\;$$ (1)

$$O_{2}+4H^{+}+4e\to 2H_{2}O\;E^{0}=4.07\;V,\;$$ (2)

$$O_{2}+2H_{2}O+4e\to 4OH^{-}\;E^{0}=3.45\;V.\;$$ (3)

A counterpart to the non-aqueous Li–air battery is the aqueous Li–air battery (Figure 1A), which utilizes an aqueous electrolyte on the cathode side and an additional lithium-ion conducting separator between the lithium anode and aqueous electrolyte to prevent lithium reaction with water (Abraham and Jiang, 1996; Imanishi et al., 2012; Imanishi and Yamamoto, 2014; Lu et al., 2014; Imanishi and Yamamoto, 2019). The lithium oxygen reaction at the cathode side is more complex than the aprotic Li–air battery and decisive to the pH (Imanishi and Yamamoto, 2014; Imanishi and Yamamoto, 2019). Under acidic conditions, oxygen reacts with hydrogen ions into water molecules based on a four-electron equation (Eq. 2), while under alkaline conditions, oxygen reacts with water into hydroxyl anions with a large difference in standard reaction potential (Eq. 3). Compared with the non-aqueous Li–air battery, the theoretical energy density of the aqueous Li–air battery is lower (1,910 Wh/kg vs. 3,505 Wh/kg) but still exceeds the practical energy density of gasoline. The major advantage of an aqueous Li–air battery over a non-aqueous system is its compatibility to be used directly in ambient air, whereas the severe water contamination from air in a non-aqueous system could be neglected. Moreover, the discharge product of LiOH is soluble in an aqueous electrolyte, which could significantly improve the areal discharge capacity and reduce the decomposition overpotential during charge. But critical challenges still remain in material stability and device performance to meet the final requirements of practical application.

In the past decade, a number of excellent books and review articles have been published regarding non-aqueous or aqueous Li–air batteries (Feng et al., 2016; Kwak et al., 2020; Dou et al., 2022; Liu et al., 2022), but the latest article for the aqueous Li–air battery has been published for more than 3 years, best to our knowledge (Imanishi and Yamamoto, 2019). As significant approaches have been made day by day, it is necessary to summarize and review them in order to accelerate the research pace for the application of aqueous Li–air batteries. In this mini-review, we focus on the current challenges of aqueous Li–air batteries relating to solid electrolytes from material to the device and provide our insight and perspective on the future development of aqueous Li–air batteries.

## Solid electrolyte separator in the aqueous Li–air battery

In the past 30 years, numerous lithium-ion-conducting solid electrolytes have emerged trying to replace traditional organic electrolytes in lithium-ion batteries. Several groups of solid electrolytes, including oxide (Aono et al., 1990; Murugan et al., 2007), sulfide (Kanno and Maruyama, 2001), halide (Steiner and Lutz, 1992), and polymer type (Tanzella et al., 1981), have been proposed to date. However, in the stage of an aqueous Li–air battery, the solid electrolyte needs to be stable to both water and oxygen, so only the oxide type of the solid electrolyte has been introduced in an aqueous Li–air battery. Back in the 2000s, Imanishi et al. studied the compatibility of oxide-type lithium aluminum titanium phosphate (LATP) (Shimonishi et al., 2011a) and lithium lanthanum zirconium oxide (LLZO) (Shimonishi et al., 2011b) in an aqueous Li–air system. Later, Inaguma et al. analyzed another kind of perovskite lithium lanthanum titanium oxide (LLTO) (Inaguma and Nakashima, 2013). All three kinds of oxide solid electrolyte showed simultaneous Li-H^+^ exchange when in contact with water or ambient air (Boulant et al., 2010; Kobi and Mukhopadhyay, 2018; Pogosova et al., 2020) as the ionic conductivity of the aged samples would be more than one order of magnitude lower than the freshly prepared ones in 1 month time. The LLZO stability was even worse as the internal stresses associated with the formation of La_2_Zr_2_O_7_ from LLZO would be high enough to cause a spontaneous fracture in the LLZO pellet, as revealed recently by Kobi and Mukhopadhyay (2018). But they were relatively stable in the saturated LiCl electrolyte or saturated LiCl with LiOH in neutral pH values less than 10 (Shimonishi et al., 2011a; Shimonishi et al., 2011b; Inaguma and Nakashima, 2013). Also, there were additional reports that LATP would be stable in the weak acidic electrolyte (Zhang et al., 2010a; Soga et al., 2020). The previous results demonstrate that LATP, LLTO, and LLZO could be suitable for aqueous Li–air batteries, but the gradually deteriorated ionic conductivity and its instability during storage in the air need further concern in the following research. Very recently, we noticed that multiple strategies, such as doping (Abrha et al., 2020; Kobi et al., 2020), coating (Yang et al., 2020; Jia et al., 2021), and ion exchange (Kim et al., 2022), were proven to be efficient in enhancing solid electrolytes toward air. Also, these may shed light on improving solid electrolyte stability in aqueous Li–air batteries.

In addition to material stability, another primary issue is the solid electrolyte weight and mechanical strength. The theoretical densities of LATP, LLZO, and LLTO are 2.93 g/cm^3^, 5.07 g/cm^3^, and 5.01 g/cm^3^, respectively, which are considerably higher than traditional organic electrolytes in the range of 1.2–1.3 g/cm^3^ and would have an adverse effect on the actual energy density of the aqueous Li–air battery. In 2014, Park et al. (2014) from Samsung Corp. provided a complex model for estimating the energy density of lithium-ion, lithium–sulfur, and lithium–air batteries as a function of areal capacity. In 2019, we further established the relationship between LATP thickness and energy density in the aqueous Li–air battery (Bai et al., 2019). As presented in Figure 1B, in order to achieve an actual energy density exceeding 300 Wh/kg in the aqueous Li–air battery, the thickness of the LAGTP solid electrolyte should be less than 100 μm. But in the early stages of the aqueous Li–air battery, the dense solid electrolyte was usually prepared by a hot-pressing technique, and the typical thickness of the electrolyte was usually larger than 500 μm, which would be detrimental to the energy density of the cell. In 2012, Takahashi et al. prepared a LATP thin sheet (about 200 μm) *via* the tape casting method. They resisted water penetration through the LATP solid electrolyte by polymerization of 2,2-bis(4-glycidyloxyphneyl) propane and 1,3-phenylenediamine in the open pores (Takahashi et al., 2012). Puech et al. (2012) further minimized free-standing LATP thickness down to 40 μm and demonstrated its potential application in aqueous Li–air batteries. But as the typical LATP mechanical strength is only around 50–80 MPa, it is still susceptible to applying such an ultra-thin pellet in an actual Li–air cell device. To reinforce the mechanical strength of thin solid electrolytes, in 2019, we introduced the secondary phase particle ceramic reinforcement strategy into the solid electrolyte (Bai et al., 2019). The as-prepared LAGTP with 10 wt% TiO_2_ exhibited a more than two-fold increase in three-point bending strength up to 200 MPa with a reduced thickness down to 90 μm (Figure 1C). Meanwhile, we systematically studied the addition of three types of transitional metal oxide to the chemo-physico-mechanical properties of the LAGTP pellet (Kyono et al., 2018). Although some achievements have been obtained in reducing solid electrolyte weight, how to improve its safety remained to be an unsolved issue. To alternatively solve solid electrolyte fragility, in 2017, Safanama et al. proposed a flexible yet dense LAGP-LiBF_4_-Butvar-PVDF-HFP composite electrolyte (Figure 1D) to be used in the aqueous Li–air battery which could significantly promote solid electrolyte practicability and cell safety (Safanama and Adams, 2017). The current issue in the composite electrolyte is that the ionic conductivity is more than one order of magnitude lower than pure solid electrolytes, which leads to a large charge–discharge overpotential in the cell. Also, its electrochemical performance degradation is much larger, which needs further study to meet the requirements in practical use. Another progress regarding the solid electrolyte weight and thickness comes from Wang et al. as they announced that the bending strength could also be relevant to the material and grain boundary properties. They compared the mechanical strengths of LLTO, LATP, and LLZO pellets prepared by tape-casting and found that the LLTO bending strength could be 2 or 16 times higher than LLZO or LATP, respectively (Jiang et al., 2020). The grain boundary composition and intra-domain control should also be important factors to be considered in the future design of solid electrolytes.

## Lithium stripping/plating based on the dense solid electrolyte

LATP is unstable against lithium metal as Ti^4+^ could easily be reduced to Ti^3+^ when contacting lithium. Therefore, a buffer layer between lithium and LATP is required to stabilize both lithium and LATP during cycling (Zhang et al., 2008; Zhang et al., 2010b). Varied by the buffer layer material type, three kinds of strategies, namely, polymer, inorganic, and liquid electrolyte, have usually been adopted to stabilize lithium plating and stripping on LATP (Wang et al., 2020b; Wang et al., 2021a; Wang et al., 2021b; Wang et al., 2022). In the early stage, poly-ethylene oxide (PEO) with lithium salt was manipulated as the buffer layer between lithium anode and LATP because it could provide a good interface contact on both materials. When lithium salt is added, lithium ions can coordinate with the polar ether oxygen group on the PEO backbone and mitigate under chain movement and electric field (Meyer, 1998). But a temperature above 60°C (glass transitional temperature) is generally needed to provide enough amorphous regions in PEO for lithium mitigation (Zhou et al., 2019). To overcome the limitation to lithium-ion conductivity by PEO, an inorganic interlayer was recently introduced to stabilize the lithium anode and its plating/stripping. For instance, Xia et al. developed a facile yet inexpensive spray-coating method to construct a three-dimensional organic/inorganic composite layer based on a commercial boron nitride-based release agent (BNRA) onto LATP (Zhu et al., 2022). The critical current density (CCD) of a symmetric Li|BNRA|LATP|BNRA|Li cell could reach 1 mA cm^−2^, which was a huge improvement in stabilizing lithium plating and stripping on the LATP pellet. In addition, other novel lithium conducting buffer layers such as succinonitrile (Cao et al., 2021) and sericin-ionic liquid (Lei et al., 2022) have also been proposed to resist electron pass from lithium to LATP.

In comparison, the progress of stabilizing Li/LLZO has been more rapid, owing to the fact that LLZO is stable toward Li. Currently, interfacial-engineered symmetric Li/LLZO/Li batteries could tolerate continuous charge–discharge for more than 1,000 h at a current density exceeding 1 mA cm^−2^. Generally, three rules have been summarized to stabilize lithium plating and stripping on the LLZO pellet. The first regulation is to diminish the surficial *in situ* formed Li_2_CO_3_. For instance, Huang et al. developed a high-speed mechanical polishing (HMP) method to remove impurities from a porous LLZTO surface and retrieve its lithiophilicity (Qin et al., 2022). Symmetric Li cells assembled with speed-controlled polishing LLZTO exhibited a critical current density exceeding 1.91 mA cm^−2^. The second regulation is to construct an electron-insulating interphase at Li/LLZO. For instance, Wen et al. built a 3D cross-linking electron-insulating LiF-LiCl network on the LLZTO surface by facile acid–salt treatment (Ruan et al., 2021). The CCDs of symmetric Li cells reached 1.8 mA cm^−2^ at 25°C (Figure 1E) and 3.6 mA cm^−2^ at 60°C. The third regulation is to resist the volume expansion between LLZO and the Li metal anode. In this perspective, He et al. designed a 3D porous Li–Zn alloy interphase (PZL) on LLZTO by the Li metal reaction with a magnetron-sputtered zinc metal layer (Wan et al., 2021). This 3D porous interphase could significantly reduce the volume expansion of the Li anode during charge–discharge. Also, the Li/LLZTO@PZL/Li symmetrical battery achieved a high critical current density of 2 mA cm^−2^. Other methods including the lithium metallic alloy and transitional metal coordination engineering by succinonitrile have also been proven to be efficient. In the next step, how to reduce LLZO thickness and improve its air stability would be essential to its application in an aqueous Li–air battery in our point of view.

At the interface between LLTO and Li, it is similar to LATP as its crystal structure is composed of Ti^4+^ which could be reduced to Ti^3+^ when contacting Li. Currently, fewer articles have been published discussing lithium plating/stripping on LLTO because of its instability toward Li and its relatively high density. However, it is worthwhile to note some novel methods to stabilize lithium utilizing the LLTO’s unique feature. For example, Ding et al. designed a mixed ionic and electronic conductor (MEIC) based on LLTO for Li–metal anode protection (Yan et al., 2018). They introduced the MIEC property in the LLTO film by using toluene as a catalyst to trigger the chemical reactions between LLTO and Li, leading to high electronic conductivity in the LLTO film. The as-fabricated Li/MEIC-LLTO/Cu could stably cycle at 0.2 mA cm^−2^.

## Catholyte design in the aqueous Li–air battery

For acidic catholytes, it provides a higher theoretical discharge potential than the alkaline counterpart in Eq. 2. Also, the side reaction with CO_2_ ingression could be significantly minimized. But to date, there is still a certain limit in acidic catholyte selection as almost all solid electrolytes are unstable to strong acids with pH values less than 5 (Manthiram and Li, 2015). Therefore, weaker acetic acid and phosphoric acid are intended to conjugate with lithium salt to adjust its pH to moderate in an aqueous Li–air battery. In 2010, Shimonishi et al. (2010) fabricated an aqueous Li–air battery with the CH_3_COOH (HAc)–H_2_O (90:10 v/v)–CH_3_COOLi (LiAc) catholyte at a pH value of 3.34. The open-circuit voltage of this cell was as high as 3.69 V as referred to in Eq. 2. But the highly volatile acetic acid could result in a low utilization efficiency of acetic acid. To deal with this issue, Zhang et al. (2010b) designed a Li/polymer electrolyte/LATP-Ohara/HAc-H_2_O-LiAc/carbon air cell to cycle under pressurized 3 atm air to suppress evaporation of the catholyte. Also, more recently, Soga et al. (2020) have modified the catholyte composition to the HAc-saturated LiAc aqueous solution (9:1 v/v), which could further promote the utilization of HAc up to 72%. But we have to admit that how to apply this catholyte for actual use remains unsolved.

Compared to the acidic catholyte, the alkaline catholyte has been demonstrated to be more efficient for ORR reaction and with higher lithium-ion conduction. To date, the LiOH base catholyte has been systematically studied. Recently, we collaborated with Suzuki Corp. to fabricate an aqueous Li–air battery with the 10 M LiCl–1.5 M LiOH catholyte that was ultra-stable to the LAGTP solid electrolyte. The pouch-type cell performed a minimum energy loss of 15% per cycle for more than 10 cycles (Bai et al., 2019). But we noted that the high concentration of inactive LiCl would inevitably lower the practical energy density of the cell and increase the potential risk of blocking the air electrode. Therefore, great efforts are still needed to improve both the catholyte efficiency and stability.

## Summary and outlooks

To summarize, current challenges in solid electrolyte separators along with related lithium plating/stripping and catholyte design are not independent but systematic in aqueous Li–air batteries. From the material level, the resistance of simultaneous Li-H^+^ exchange between the catholyte and solid electrolyte should be improved as a first priority as it is still suffering from the long-term instability of the solid electrolyte in a semi-open cell structure. Methods such as element doping, grain-boundary regulation, and surface modification of the solid electrolyte could be feasible to alleviate this problem.

From the battery level, the weight of solid electrolytes should be further concerned. A feasible guideline for minimizing weight, while not reducing mechanical strength, is to design a polymer–ceramic composite electrolyte for the aqueous Li–air battery. But the catholyte and lithium anode corrosion to the composite electrolyte, especially the polymer part, should be solved initially before being utilized in fabricating a battery.

Regarding the lithium stripping/plating relating to a solid electrolyte or composite electrolyte, a buffer layer between the solid electrolyte and lithium needs to be carefully designed to consider possible dendrite growth pathways or reduction reactions based on various kinds of the solid electrolyte. For example, buffer layers based on solid electrolytes should be electron-blocking when the solid electrolyte contains typical transitional metal ions like Ti^4+^ and Ge^4+^, but it would be good when contacting LLZO.

Furthermore, the catholyte design should also consider the properties of solid electrolytes. To date, deep charge–discharge is seldomly concerned in the published articles, but the violently changed charge–discharge product concentration would inevitably influence the catholyte pH and further influence the solid electrolyte separator. The mechanism behind it also needs to be thoroughly investigated at both molecular and macroscopic levels.

Last but not least, from our point of view, the cell configuration also demands further investigation. Currently, Swagelok-type cells with heavy steel frames or pouch-type cells with simple stacking could not meet the requirement of both high energy density and battery safety. To minimize frame weight while maximizing the air utilization efficiency, a lighter weight frame such as aluminum mesh with a designed air diffusion channel should be considered in the future study.

Although great challenges are still ahead on the road to aqueous Li–air battery industrialization, we are confident that the rapid innovation in material science and engineering would undoubtedly bring prospects to the application of aqueous Li–air batteries in the near future.
